# The Burden of the Pain: Adverse Mental Health Outcomes of COVID-19 in Women With and Without Cancer

**DOI:** 10.3389/fpsyg.2022.772128

**Published:** 2022-04-05

**Authors:** Lucilla Lanzoni, Eleonora Brivio, Serena Oliveri, Paolo Guiddi, Mariam Chichua, Ketti Mazzocco, Gabriella Pravettoni

**Affiliations:** ^1^Applied Research Division for Cognitive and Pychological Science, Istituto Europeo d’Oncologia IRCCS, Milan, Italy; ^2^Department of Oncology and Hemato-Oncology, University of Milan, Milan, Italy

**Keywords:** COVID-19, cancer, post-traumatic stress, generalized anxiety, psychological outcomes, female health

## Abstract

The coronavirus disease 2019 (COVID-19) pandemic had a negative psychological impact on the population at scale, yet it is possible that vulnerable patient populations may experience a heavier burden with increased feelings of anxiety and distress. Cancer patients have to trade-off between the fear of exposing themselves to the virus and the need to continue life-saving medical procedures. The present study investigated the prevalence of generalized anxiety and post-traumatic stress symptoms (PTSS) in a population of Italian cancer patients and healthy participants in the months following the COVID-19 outbreak. Using standardized measures of PTSS (impact of event scale revised; IES-R) and generalized anxiety (generalized anxiety scale; GAD-7), we found that patients experienced higher levels of adverse mental health outcomes. Several variables were found to negatively affect PTSS and anxiety in this population, including the younger age of respondents, having children, and the impossibility to attend regular medical check-ups. These findings stress the importance of maintaining a clear and regular communication with patients throughout future waves of the pandemic and ensure continuity of care in this vulnerable population. Furthermore, this study indicates the need to establish psychological interventions aimed at patients with cancer, targeting especially younger generations who are more likely to experience adverse psychological outcomes.

## Introduction

The coronavirus disease 2019 (COVID-19) outbreak originated in Wuhan, China in late December 2019 and quickly escalated into a global pandemic (Liu et al., 2020; Zhu et al., 2020). While this has caused formidable disruption to the world population at scale, the psychological impact in vulnerable groups with pre-existing conditions calls for special attention. As a life-threatening disease, cancer increases the risk of developing mental health problems, including depression, anxiety, and distress (Mitchell et al., 2011; Inagaki et al., 2015; Ng et al., 2018). It is estimated that approximately one-third of cancer patients are affected by mental health disorders (Massie, 2004; Singer et al., 2010; Mitchell et al., 2011). Additionally, at the time of the study, it was unclear whether the effects of COVID-19 would be more severe in cancer patients. The general consensus is now that the mortality rate for infected cancer patients is significantly higher compared to the general population (Chen et al., 2020a; Gosain et al., 2020; Mehta et al., 2020). Thus, in a situation of health emergency, such as the one we are currently facing, these patients are likely to experience a trade-off between the increased risk of contracting COVID-19 when receiving cancer treatments and the increased risk of worsening cancer prognosis when postponing treatments (Burki, 2020). The present study explores the prevalence of COVID-19-related distress and anxiety in a population of Italian cancer patients.

A growing number of studies conducted during the COVID-19 pandemic have already highlighted mental health issues in the general population (Cuiya et al., 2020; Hossain et al., 2020a,b; Xiong et al., 2020). In Italy, one of the most severely hit countries in Europe in the first wave of the pandemic, several studies have explored the psychological impact of the pandemic using online and remotely administered surveys (Ferrucci et al., 2020; Mazza et al., 2020; Rossi et al., 2020; Brivio et al., 2021a). Three weeks into the national lockdown Rossi et al. (2020) found relatively high levels of adverse mental health outcomes among Italians, with 37% reporting post-traumatic stress symptoms (PTSS), 17% reporting depressive symptoms, and 21% reporting severe anxiety symptoms. Being a woman and younger age were associated with more severe psychological outcomes. Focusing on the COVID-19 pandemic as a traumatic event, partly worsened by media communication (Brivio et al., 2020), Brivio et al. (2021a) assessed PTSS and generalized anxiety in the months following the disease outbreak. An online survey was set up for this exploratory study and included the standardized Italian versions of the impact of event scale revised (IES-R) and the generalized anxiety scale (GAD-7), as well as socio-demographic questions to identify potential risk factors. Results revealed severe levels of PTSS and mild levels of anxiety in the general population and identified important gender and age differences. Specifically, younger respondents displayed higher levels of distress and anxiety than older respondents, and female respondents experienced greater distress and anxiety compared to males. This gender effect mirrors findings from other studies (Mazza et al., 2020; Rossi et al., 2020) and appears to be consistent with studies on the fear of COVID-19, where anxiety is observed to be higher in women (Evren et al., 2020; Soraci et al., 2020; Reznik et al., 2021).

COVID-19 has detrimental effects on the lives of cancer patients due to the uncertainty regarding treatment and screening, combined with the fear of being infected (Gregucci et al., 2020; Brivio et al., 2021b). Several studies have reported considerably high levels of anxiety, depression, and post-traumatic stress disorder (Beaty et al., 2018; Chen et al., 2020b, 2021; Sigorski et al., 2020; Swainston et al., 2020; Ayubi et al., 2021; Yasin, 2021), and increased emotional vulnerability (Pigozzi et al., 2021). In some cases, respondents were cancer patients undergoing treatment at the time of the study (Sigorski et al., 2020; Chen et al., 2021), but active treatment status was not always among the inclusion criteria (e.g., Swainston et al., 2020; Yasin, 2021). The vast majority of surveys were administered online and assessed patients’ psychological health (Chen et al., 2020b; Romito et al., 2020; Sigorski et al., 2020; Swainston et al., 2020; Yasin, 2021) and their fear of tumor progression (Chen et al., 2020b; Sigorski et al., 2020) during the first wave of the COVID-19 pandemic. Work from China revealed that among 6,213 cancer patients, 23.4% had depression, 17.7% had anxiety, and 9.3% had post-traumatic stress disorder (Wang et al., 2020). Compared with males, females had a higher frequency of worrying about disease management, higher psychological pressure, and lower sleep quality because of the pandemic. In Italy, Romito et al. (2020) used validated questionnaires of anxiety, depression, and PTSS in patients with lymphoma and found that 36% fulfilled the diagnostic criteria for anxiety, 31% for depression, and 36% scored above cutoff for PTSS. In line with other studies, women were found to be more vulnerable to both anxiety and PTSS (Dückers and Olff, 2017), while younger patients showed higher levels of PTSS, an effect which mirrors the one seen in healthy participants (Rossi et al., 2020; Brivio et al., 2021a).

At present, only a handful of studies have directly compared the psychological effects of the pandemic on cancer patients and healthy controls, and the evidence is not always consistent (Musche et al., 2020; Ng et al., 2020). Here we contrasted the levels of anxiety and PTSS in cancer patients and healthy participants using two standardized tools, namely, the Generalized Anxiety Disorder (GAD-7) and the Impact of Event Scale revised (IES-R). Building on the literature showing an increased risk of anxiety symptoms and stress in women in both cancer (Romito et al., 2020; Sigorski et al., 2020) and healthy populations (Dückers and Olff, 2017; Rossi et al., 2020; Brivio et al., 2021a), this investigation focused on a female sample. Disruption to treatment and screening was also considered, given previous evidence of higher emotional vulnerability in women who experienced disruptions to scheduled oncology services (Swainston et al., 2020). The aim of the present study was twofold. First, we aimed to compare the prevalence of generalized anxiety and PTSS between cancer patients and healthy respondents in Italy, in the months following the outbreak. Given the additional burden that patients with cancer are experiencing, we expected to find higher adverse psychological outcomes in patients compared to healthy participants that are matched by socio-demographic variables. To our knowledge, this is the first study to investigate the topic in Italian cancer patients using a differential design. Second, having established the extent of the psychological impact of the pandemic on cancer patients, the study further examined if socio-demographic variables which have previously been correlated to psychological outcomes in the general public might be associated with psychological adverse events in our cancer population.

## Materials and Methods

### Procedure

Data collection took place in the first wave of the COVID-19 pandemic, between April and June 2020. The recruitment strategy used a snowball sampling method, which is a type of chain-referral non-probabilistic sampling technique involving the recruitment of new participants by current study subjects (Lewis-Beck et al., 2003; Ghaljaie et al., 2017). The survey was hosted on Qualtrics, and anonymous links were generated to be distributed among the population. Healthy participants received the link *via* the social media accounts (WhatsApp, Facebook, and Instagram) of the authors. For the cancer population, the survey link was shared across different newsletters and social media (Facebook and Instagram) pages of several Italian patient associations. Snowball sampling methods with online surveys have been used in several studies investigating mental health issues during the COVID-19 pandemic (Fekih-Romdhane et al., 2020; Rapisarda et al., 2020; Sharma and Vaish, 2020; Arafa et al., 2021), as they offer an important advantage in a situation of confinement due to the outbreak. Respondents were considered eligible if they resided in Italy at the time of the COVID-19 pandemic and if they were at least 18 years of age and Italian native speakers. If they did not meet these inclusion criteria, they were automatically taken to the last page of the survey. The complete dataset for the healthy participants has been published in Brivio et al. (2021a).

### Measures

The survey comprised questions about socio-demographic and health variables, as well as standardized measures of psychological distress and anxiety (Spitzer et al., 2006; Weiss, 2007). Socio-demographic and health variables covered information about age, gender, education level, marital and parental status of healthy participants and cancer patients. Group-specific information was also gathered: patients were probed with questions about the year of cancer diagnosis, and the status of their medical treatments (“no treatment,” “treatment suspended,” and “treatment maintained”) and screening protocols (“no medical screening,” “medical screening suspended,” and “medical screening maintained”) at the time of the survey, while healthy participants were asked about the presence of a chronic disease. The impact of event scale revised (IES-R) in its validated Italian version (Craparo et al., 2013) and the Generalized Anxiety Disorder scale (GAD-7; Spitzer et al., 2006) were used to assess symptoms of post-traumatic stress disorder (PTSS) and generalized anxiety, respectively. The IES-R comprises 22 items divided into three subscales measuring intrusion (IES-RI: eight items), avoidance (IES-RA: eight items), and hyperarousal (IES-RH: six items) symptoms of post-traumatic stress, rated on a 4-point Likert scale from 0 (not at all) to 4 (extremely). A total raw score of 24 or above is a clinical concern, with values between 24 and 32 suggesting mild PTSS, 33–36 for moderate PTSS, and > 37 for severe PTSS (Creamer et al., 2003; Weiss, 2007; Rash et al., 2008). The GAD-7 has seven items for frequency of symptoms over the previous 2 weeks measured on a 4-point Likert scale and combined into a total sum score (Spitzer et al., 2006). Scores of 5, 10, and 15 were used as the cutoff points for mild, moderate, and severe anxiety.

### Participants and Data Quality Assessment

Out of a total of 1,743 respondents (544 patients, 1,199 healthy), 464 participants (280 patients, 184 healthy; see Table 1 for sample characteristics) were carried forward into the analyses following a conservative procedure of data quality assessment and matching. This was performed in subsequent steps. First, respondents who had failed to respond to IES-R questions were removed, as the psychological effects of the COVID-19 outbreak were the focus of this study. Next, with over 86% of cancer patients identifying themselves as female and less than 1% belonging to generation Z, male respondents (regardless of age) and generation Z respondents were removed from both the clinical and control samples. Moreover, healthy respondents with a chronic condition were discarded to avoid possible biases associated with suffering from an illness. In a third step, we aimed to match the two samples based on socio-demographic variables using a stepwise procedure. Specifically, for each generation group (i.e., baby boomers, generation X, and generation Y), and for each level of education (i.e., compulsory, higher education, and university degree) within a given generation group, we calculated patients’ mean age. Healthy participants falling outside the ±1 standard deviation range were excluded.

**Table 1:** Socio-demographic, clinical, and psychological variables.

Variables	Levels	Oncological patients	Healthy controls
		*N* (%)	*N* (%)
*Socio-demographic and clinical variables*			
Years since diagnosis: mean [SD]		5.1 [5.5]	
Age: mean [SD]		50 [9.4]	48.2 [9.6]
Age generations	*Baby boomers*	93 (33.2)	60 (32.6)
	*Gen X*	145 (51.8)	83 (45.1)
	*Gen Y*	42 (15)	41 (22.3)
Education level	*Compulsory education*	25 (8.9)	10 (5.4)
	*Secondary school*	130 (46.4)	70 (38)
	*University degree*	124 (44.3)	104 (56.5)
Marital status	*Without partner*	107 (38.2)	76 (41.3)
	*With partner*	171 (61.1)	108 (58.7)
Parental status	*Without children*	99 (35.4)	67 (36.4)
	*With children*	180 (64.3)	117 (63.6)
Cancer treatment	*No*	141 (50.4)	
	*Yes and it was interrupted*	3 (1.1)	
	*Yes and it was maintained*	136 (48.6)	
Medical screening	*No*	54 (19.3)	
	*Yes and it was interrupted*	88 (31.4)	
	*Yes and it was maintained*	114 (40.7)	
*Psychological variables*			
Post-traumatic stress disorder (IES-R)	*No PTSD (*≤ 23)	58 (20.7)	74 (40.2)
	*Mild PTSD (24–32)*	45 (16.1)	32 (17.4)
	*Moderate PTSD (33–36)*	32 (11.4)	21 (11.4)
	*Severe PTSD (≥ 37)*	145 (51.8)	57 (31)
Generalized anxiety disorder (GAD-7)	*No clinical anxiety (≤ 4)*	48 (17.1)	63 (34.2)
	*Mild anxiety (5–9)*	103 (36.8)	76 (41.3)
	*Moderate anxiety (10–14)*	69 (24.6)	29 (15.8)
	*Severe anxiety (≥ 15)*	50 (17.9)	16 (8.7)

### Analytic Strategy

To reduce the complexity of the dataset and facilitate interpretation, several variables were recoded. Age was grouped according to generation: baby boomers, born between 1946 and 1965; generation X, born between 1966 and 1980; generation Y (or millennials), born between 1981 and 1995. Education was recoded into three levels: compulsory education, including elementary and middle school diploma; secondary school, or high school diploma; and university degree, including bachelor and postgraduate studies. Marital status was recoded into two levels: without a partner, including widows, divorced, separated, or singles; and with partner, married, or in a significant relationship.

Analyses were performed on IBM SPSS v. 26. First, we used non-parametric Chi^2^ to ensure that healthy participants and cancer patients did not differ significantly in terms of socio-demographic variables. Next, differences between patients and healthy respondents in the levels of PTSS and anxiety were analyzed using parametric tests, including MANOVA, ANOVA, and *t*-tests. Finally, the effect of socio-demographic and clinical variables on patients’ scores on IES-R and GAD-7 were analyzed using non-parametric Kruskal–Wallis’ test (H) and Mann–Whitney’s test (U), since the variables did not satisfy the assumptions of normality and homogeneity of variance, and the groups had largely different sample sizes. Wherever multiple comparisons were performed, a Bonferroni correction was applied.

## Results

### Socio-Demographic Information

Sample characteristics can be found in Table 1. Cancer patients and healthy participants did not differ in terms of age [*χ*^2^ (2) = 4.3, *p* = 0.116], marital status [*χ*^2^ (1) = 0.37, *p* = 0.545], and parental status [*χ*^2^ (1) = 0.04, *p* = 0.838]. The model comparing levels of education across the two groups was significant [*χ*^2^ (3) = 6.99, *p* = 0.030]; however, a closer inspection of the standardized residuals (all within +/− 1.96) revealed no significant differences between conditions.

### Comparison of Patients and Healthy Participants

**Figure 1:**
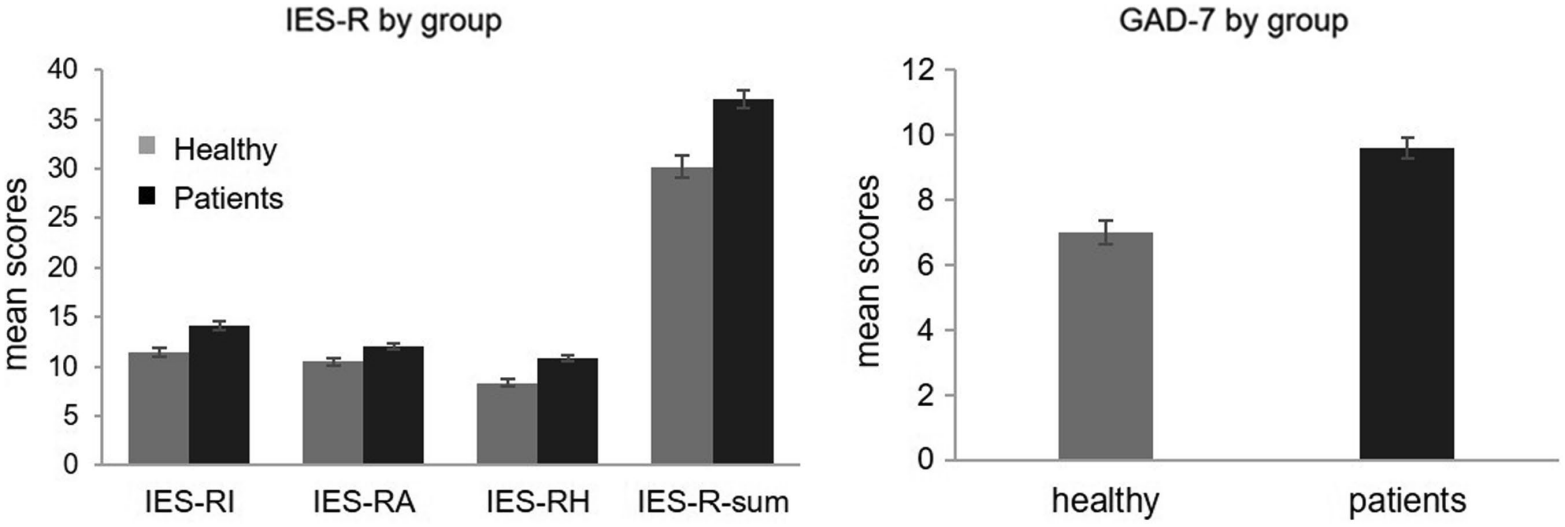
Bar chart showing the mean scores for oncological patients (black) and healthy participants (grey) in IES-R (left) and GAD-7 (right).

The mean scores for cancer patients and healthy participants on the two measures of interest are displayed in Figure 1.

#### Post-traumatic Stress Symptoms

Cancer patients showed severe levels of PTSS, with a total IES-R mean score of 37.1 (SD = 14.7), while healthy respondents had a mean score of 30.2 (SD = 14.8), indicative of mild levels of distress. Having established that the data met the general assumptions for parametric test statistics as well as the assumptions for multivariate tests, group differences in the scores obtained on the three IES-R subscales were analyzed with a MANOVA. Using Pillai’s trace, a significant effect of the group was found [V = 0.06, *F*(3,460) = 8.9, *p* < 0.001, *η*^2^ = 0.06]. *Post-hoc* analyses with separate univariate tests on the IES-R dimensions revealed significant differences between groups on intrusion [*F*(1,462) = 21.1, *p* < 0.001, *η*^2^ = 0.04], avoidance [F(1,462) = 9.1, *p* = 0.002, η^2^ = 0.02], hyperarousal [F(1,462) = 26.6, p = <0.001, η^2^ = 0.05], with cancer patients scoring higher than healthy participants on all dimensions.

#### Symptoms of Generalized Anxiety

Ten patients did not answer GAD-7 questions and were therefore not included in the analyses. The mean GAD scores for both patients (*M* = 9.6, SD = 5.2) and healthy participants (*M* = 7, SD = 4.8) were indicative of mild anxiety. An independent t-test revealed that patients had significantly higher levels of anxiety compared to healthy respondents [t(414.1) = −5.5, *p* < 0.001].

### Exploration of Psychological Distress and Anxiety in the Patient Sample

As described in the previous section, group comparisons revealed significant differences in the two psychological constructs of interest, with patients experiencing higher levels of distress and anxiety compared to healthy participants. In a further analysis, we used non-parametric tests to explore associations between mental health outcomes in cancer patients and different socio-demographic and clinical variables, such as age, level of education, marital and parental status, and the possibility to continue medical visits (while the possibility to continue cancer therapy could not be investigated due to a small numerosity of the subgroup). Median and interquartile range values are reported in Table 2.

**Table 2:** Median and (interquartile range) values for IES-R and GAD-7 items.

		IES-R Intrusion	IES-R Avoidance	IES-R Hyperarousal	IES-R sum score	GAD-7 sum score
		Mdn (IQR)	Mdn (IQR)	Mdn (IQR)	Mdn (IQR)	Mdn (IQR)
Age generations	*Baby boomers*	14 (10)	12 (7)	9 (8)	35 (23)	7 (8)
	*Gen X*	14 (9)	12 (6)	10 (7)	36 (18)	9 (7)
	*Gen Y*	15 (10)	13 (6)	13 (6)	40 (20)	9 (7)
Education level	*Compulsory education*	14 (13)	12 (8)	13 (9)	37 (20)	11 (7)
	*Secondary school*	15 (10)	11 (6)	11 (8)	36.5 (25)	9 (7)
	*University degree*	14 (8)	12 (6)	10 (7)	37 (17)	8 (7)
Marital status	*Without partner*	14 (9)	12 (5)	10.5 (8)	36 (19)	8 (9)
	*With partner*	15 (10)	12 (7)	10 (8)	38 (21)	9 (7)
Parental status	*Without children*	14 (9)	12 (6)	11 (8)	36 (22)	9 (8)
	*With children*	15 (10)	12 (6)	10 (8)	37 (21)	8.5 (7)
Medical screening	*No*	14 (9)	13 (7)	10 (9)	34 (22)	9 (8)
	*Yes and it was interrupted*	16 (7)	12 (6)	13 (8)	41 (17)	10 (7)
	*Yes and it was maintained*	12 (9)	11 (6)	9 (7)	34 (20)	7 (7)
Total (N)			280			270

All test statistics are reported in Table 3. Independent-samples Kruskal–Wallis tests (H) revealed significant effects of age generation on the hyperarousal dimension of PTSS [H(2) = 7.71, *p* = 0.021], with generation Y respondents scoring higher than baby boomers on the hyperarousal subscale [H(1) = −41.7, *p* = 0.017]. Age generation had a similar effect on the levels of anxiety [H(2) = 10.9, *p* = 0.004; experienced during the pandemic, with generation Y respondents showing higher generalized anxiety than baby boomers [H(1) = −46.27, *p* = 0.005]. No significant results were observed for the level of education of participants.

**Table 3:** Values for the non-parametric statistics.

		IES-R Intrusion	IES-R Avoidance	IES-R Hyperarousal	IES-R sum score	GAD-7 sum score
Age generations	H	1.703	1.668	7.707	4.082	10.905
	df	2	2	2	2	2
	*p*	0.427	0.434	**0.021**	0.13	**0.004**
Education level	H	0.952	4.459	2.059	0.692	5.633
	df	2	2	2	2	2
	*p*	0.621	0.108	0.357	0.708	0.06
Marital status	U	9,942	9,697	9,732	9,980	8,360
	z	1.218	0.844	0.896	1.275	−0.271
	*p*	0.223	0.399	0.37	0.202	0.786
Parental status	U	10,376	9,118	9,595	9,984	8,636
	z	2.276	0.323	1.065	1.666	0.544
	*p*	**0.023**	0.746	0.287	0.096	0.586
Medical screening	H	20.436	13.598	18.591	23.214	17.745
	df	2	2	2	2	2
	*p*	**<0.001**	**0.001**	**<0.001**	**<0.001**	**0.001**

Significant values are displayed in bold.

Parental status was found to explain differences in the intrusion dimension of PTSS. An independent-sample Mann–Whitney U-test (U) revealed that patients with children had more frequent intrusions than patients without children (*U* = 10.38, *z* = 2.28, *p* = 0.023). In contrast, marital status (or more generally the presence of a partner) did not yield significant results.

The variable “medical screening” was significantly associated with all three dimensions of PTSS [IES-RI: H(2) = 20.44, *p* < 0.001; IES-RA: H(2) = 13.6, *p* = 0.001; IES-RH: H(2) = 18.59, *p* < 0.001]. Compared with patients who continued to be regularly screened, patients whose medical check-ups were interrupted had higher intrusion, hyperarousal and avoidance scores (IES-RI: *H* = 47.09, z = 4.5; *p* < 0.001; IES-RA: *H* = 38.36, *z* = 3.66, *p* < 0.001; IES-RH: *H* = 44.62, *z* = 4.25, *p* < 0.001). Additionally, these patients scored higher in both intrusion and hyperarousal dimensions compared to patients who were not attending medical visits prior to the outbreak (IES-RI: *H* = −32.76, *z* = −2.56; *p* = 0.031; IES-RH: *H* = −33.14, *z* = −2.6, *p* = 0.029). A similar effect of medical check-ups was found for GAD-7 [H(2) = 17.75, *p* = 0.001], whereby respondents who could not continue their medical visits during the pandemic experienced significantly higher levels of anxiety compared to patients who continued to have regular medical check-ups (*H* = 39.02, *z* = 3.8, *p* < 0.001).

## Discussion

Feelings of anxiety and distress are a common feature of situations of uncertainty, of which the COVID-19 pandemic is a recent example. While the health crisis has affected mental health globally, cancer patients are more likely to experience contrasting emotional states, battling between the need to continue life-saving medical procedures and the fear of exposing themselves to the virus. The present study investigated the psychological burden of cancer patients in Italy in the months following the outbreak. We contrasted psychological outcomes in a sample of cancer patients and a group of healthy respondents matched for age and education using the same validated measures of generalized anxiety and PTSS. Results revealed higher levels of anxiety and PTSS in patients compared to healthy participants. In the cancer population, several clinical and demographic variables were found to affect the probability of developing adverse mental health outcomes. In particular, younger patients experienced higher levels of hyperarousal and anxiety compared to older patients, while respondents with children had more frequent intrusions than those without children. The impossibility to attend regular medical visits throughout the lockdown was found to be a risk factor, with patients whose appointments were postponed experiencing higher levels of PTSS and anxiety. Overall, these findings are in line with previous evidence collected during the pandemic and highlight the importance of monitoring mental health in vulnerable cancer populations.

While control groups of healthy respondents have sometimes been used in similar studies, the evidence is scarce and not always consistent. For example, Musche et al. (2020) showed comparable levels of distress and general anxiety in treated cancer patients relative to healthy controls. Another study found low levels of psychological distress and anxiety in both cancer survivors and healthy controls, with significantly lower levels of distress in the cancer population (Ng et al., 2020). Such pattern of results might be explained in terms of more effective coping strategies in cancer patients, who have learned to deal with stressful situations effectively (Livneh, 2000), but they may also be indicative of a low psychological distress and anxiety across groups. In contrast, our study showed elevated levels of distress and anxiety in response to the health emergency. This difference may be partially due to the way communication was initially managed at mass level during the first wave of COVID-19 (Brivio et al., 2020), when the risk of infection in Italy was profoundly different from that experienced in other European countries, where the other studies were conducted.

In this framework, the novel findings that oncological patients experienced more severe PTSS and anxiety than healthy participants can be explained assuming the COVID-19 pandemic as a source of trauma that would particularly affect individuals who are already physically and psychologically vulnerable due to cancer (Miaskowski et al., 2020). In this context, adverse mental health outcomes may be the result of overthinking about their safety and feeling in danger of contracting the virus, because of their physical debilitation and a reduced immune response due to cancer treatments. Future studies could explore associations between the subjective level of information about COVID 19 and adverse mental health outcomes, as high levels of information may reduce the psychological burden experienced by patients and level down differences with the healthy population (Musche et al., 2020).

Unlike previous studies which have assessed PTSS using the IES-R total sum score (Fiorillo and Gorwood, 2020; Romito et al., 2020; Wang et al., 2020; Joly et al., 2021), here we focused on the different sub-dimensions of post-traumatic stress disorder. This becomes interesting in the exploration of socio-demographic and clinical variables associated with negative mental health outcomes in cancer patients (Oliveri et al., 2019). We showed that younger respondents (aged 26–40) experienced more hyperarousal symptoms and anxiety than older patients (aged 56–75). These results replicate recent evidence by Brivio et al. (2021a) and are consistent with previous literature showing that younger age groups tend to have higher levels of PTSS than senior respondents (Ozamiz-Etxebarria et al., 2020; Romito et al., 2020), and experience greater cancer-related distress compared to older patients (Burgoyne et al., 2015). During the pandemic, this group had to delay major life milestones, such as buying a home, planning a family, or making a career move. We should also consider that many women diagnosed with cancer undergo treatment while negotiating work and family responsibilities, and this is particularly true for women belonging to generation Y. Nevertheless, the age difference could also be suggestive of a life span pattern of distress, whereby distress decreases with age independently of cancer or other chronic illnesses (Chittleborough et al., 2011). Among the socio-demographic variables examined, parental status was also associated with PTSS. Specifically, patients with children had more frequent intrusions compared to patients without children, possibly reflecting a heightened sense of responsibility toward the family in the event of contracting the disease. This result resonates with research showing greater chance of developing anxiety and depression in cancer patients with children as compared to similarly ill patients without cancer (Nilsson et al., 2009).

Additionally, analyses of the dimensions of IES-R showed that having to pause medical visits caused a significant increase in PTSS and anxiety. While we did not differentiate between cancer types in our study, future research should explore whether specific cancers (i.e., those with a faster progression rate) may be associated with different levels of anxiety and PTSS. Nevertheless, this finding is consistent with the literature (Swainston et al., 2020) and suggests that patients fear the consequences associated with interrupting their regular contacts with the hospital. Such insights are relevant for the management of hospital distress related to the COVID-19 pandemic, with psychological interventions becoming core components of cancer care protocols (De Berardinis et al., 2021). Future studies could also explore the psychological impact that COVID-19 pandemic had on patients who were undergoing different types of therapies (e.g., chemotherapy, radiotherapy, immunotherapy, and adjuvant hormone therapy) to explore differences in distress levels and specific needs.

A final consideration concerns previously documented gender differences in mental health, where mounting evidence suggests that women are more vulnerable to anxiety and distress. In a recent study, Brivio et al. (2021a) found higher levels of stress during the COVID-19 pandemic in female compared to male participants, replicating similar studies (Pappa et al., 2020; Vindegaard and Benros, 2020). Here we focused on female cancer patients, who are more likely to suffer the psychological burden of COVID-19. Nevertheless, our direct comparison of cancer and non-cancer patients allowed us to isolate the effect of being a female cancer patient during the pandemic. A large number of women have lost their job, been put on leave, or have reduced their hours due to the pandemic, causing financial stress that amplified the level of distress generated by the life-threatening disease (Simha et al., 2020). Future studies could explore interactions between gender and clinical interventions aimed at reducing COVID-19-related anxiety.

The primary strength of this study is that the empirical evidence used to compare the two populations was generated using the same standardized measures, allowing for direct comparison of results. The observation that patients with cancer experience more adverse mental health outcomes stresses the importance of providing continuity of care throughout future waves of the pandemic, and highlights the role of a clear and honest communication about the strategies to mitigate contagion risks. This study also had some limitations. First, the generalizability of the results is limited by the sampling technique adopted, which may have introduced biases in the sample composition. Second, the surveys were administered online, with respondents being given instructions as to how to complete the survey. While this gave us the possibility of reaching a wider geographical area, a general limitation of online research is the lack of control over who is completing the survey. It is unclear whether respondents received help when completing the survey, and how this may have influenced their answers.

In conclusion, this research highlights the importance of providing psychological interventions and support to cancer patients in pandemic times. With technology advancing at unprecedented rates, e-health applications could be used as valid alternative to guarantee a continuous communication with patients. This may be particularly beneficial for younger generations, who are not only the ones experiencing higher distress and anxiety, but also those who may be more favorable to digital medicine. Further research is required to identify the most effective channels and devices.

## Data Availability Statement

The raw data supporting the conclusions of this article will be made available by the authors, without undue reservation.

## Ethics Statement

The studies involving human participants were reviewed and approved by the European Institute of Oncology Ethical Committee. The patients/participants provided their written informed consent to participate in this study.

## Author Contributions

EB, SO, PG, KM, and GP contributed to conception and design of the study. LL, EB, and MC organized the database and performed the statistical analysis. LL wrote the initial draft of the manuscript and revised the drafts during peer-review. GP supervised the study. All authors contributed to the article and approved the submitted version.

## Funding

EB is supported by a personal grant from Fondazione Umberto Veronesi.

## Conflict of Interest

The authors declare that the research was conducted in the absence of any commercial or financial relationships that could be construed as a potential conflict of interest.

## Publisher’s Note

All claims expressed in this article are solely those of the authors and do not necessarily represent those of their affiliated organizations, or those of the publisher, the editors and the reviewers. Any product that may be evaluated in this article, or claim that may be made by its manufacturer, is not guaranteed or endorsed by the publisher.
